# Synchronization of kinetic and kinematic hand tasks with electrocorticography and cortical stimulation during awake craniotomies

**DOI:** 10.1371/journal.pone.0283460

**Published:** 2023-03-27

**Authors:** Léon Taquet, Brian J. Conway, Timothy F. Boerger, Sarah C. Young, Stephanie Schwartz, Brian D. Schmit, Max O. Krucoff

**Affiliations:** 1 Medical College of Wisconsin, Milwaukee, WI, United States of America; 2 Department of Neurosurgery, Medical College of Wisconsin, Milwaukee, WI, United States of America; 3 Department of Biomedical Engineering, Marquette University, Milwaukee, WI, United States of America; Belgrade University Faculty of Medicine, SERBIA

## Abstract

Awake craniotomies provide unique and invaluable scientific opportunities for neurophysiological experimentation in consenting human subjects. While such experimentation carries a long history, rigorous reporting of methodologies focusing on synchronizing data across multiple platforms is not universally reported and often not translatable to across operating rooms, facilities, or behavioral tasks. Therefore, here we detail an intraoperative data synchronization methodology designed to work across multiple commercially available platforms to collect behavioral and surgical field videos, electrocorticography, brain stimulation timing, continuous finger joint angles, and continuous finger force production. Our technique was developed to be nonobstructive to operating room (OR) staff and generalizable to a variety of hand-based tasks. We hope that the detailed reporting of our methods will support the scientific rigor and reproducibility of future studies, as well as aid other groups interested in performing related experiments.

## Introduction

There is a long, rich history of intraoperative neurophysiological experimentation leading to advances in the understanding of neurophysiology and neuroanatomy that continues today [1]. Specific to hand motor physiology, for example, several groups have recently quantified positive and negative motor responses to stimulation of the precentral gyrus when flexing/extending upper limbs and motor suppression during a knob turning task with stimulation of the precentral gyrus [2, 3]. While there is a lot of published data from these types of intraoperative studies, there is minimal published literature focusing on the details of how to collect and synchronize data across disparate platforms while integrating them into an awake brain operation [2]. This is critical to ensuring scientific rigor and reproducibility of the results, as well as practicality and comfort for the participant and operating room staff. Therefore, our purpose is to outline a general, repeatable methodology for cross-platform data collection and synchronization of brain and hand signals using commercially available products.

**Fig 1:**
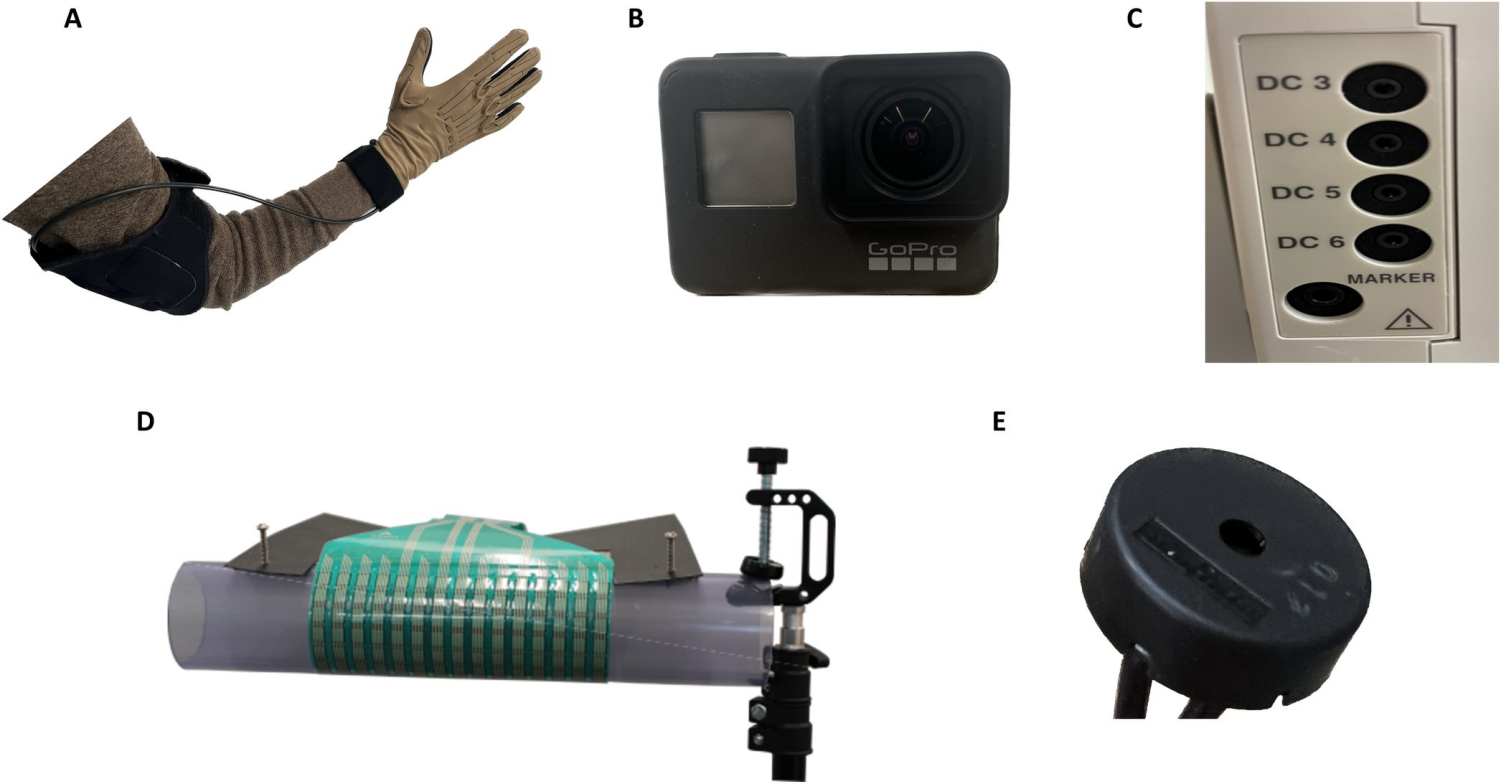
Intraoperative hand kinematics and kinetic recording devices. (A) CyberGlove III uses strain gauges and patient-specific calibrations to provide angle measurements for 23 different joints at 90 Hz. (B) A stock GoPro Hero 7 Black is used to record patient hand movements, and a custom GoPro Hero 7 Black with 8.25mm (47mm) f/2.8 lens for recording the surgical/stimulation site. (C) Nihon Kohden EEG-1200AA series is used clinically to monitor for seizure activity via ECoG grids near stimulation areas and allows for the input of parallel DC channels for other devices. (D) TekScan F-Socket pressure mat affixed to a 2.5 in diameter PVC pipe provides force recordings of hand grips at 200 Hz from force sensors at a density of 0.7 sensors/cm^2^. (E) Piezoelectric sensor outputs voltage differences across its terminals recorded by a Nihon Kohden DC channel in response to loud sounds such as the Go/Stimulate tone.

Specifically, we will demonstrate how to synchronize whole hand kinematics, kinetics, direct cortical stimulation (DCS), electrocorticography (ECOG), surgical video, and task video in a generalizable manner. We further explore the limitations and characterize lags between several commercially available devices, such as the high-resolution force mat (F-Socket TekScan) and a 23-joint-angle (DoF) CyberGlove III data glove for hand tracking, a standard clinical EEG (Nihon Kohden EEG-1200AA) for intraoperative ECoG, a customized GoPro Hero 7 Black with 8.25mm (47mm) f/2.8 lens (PeauProductions) for surgical and behavioral video, and a custom synchronization platform that uses a reference transistor-transistor logic (TTL) voltage for cross-platform alignment (the design of which is shared here) (Fig 1). Ideally, the general framework of this synchronization protocol can be expanded in future protocols to include other intraoperative motor tasks, or even tasks with other modalities, like speech.

## Materials and methods

The protocol described in this peer-reviewed article is published on protocols.io, DOI: dx.doi.org/10.17504/protocols.io.5jyl89638v2w/v1 (Private link for reviewers: https://www.protocols.io/private/A4156E7AB8C0D9AA933F36858AC3EF3D to be removed before publication.) and is included for printing as supporting information file 1 with this article.

The protocol was developed as part of a study approved by the Medical College of Wisconsin and Froedtert Hospitals IRB with the identifier PRO00040251. Written consent was received by participants.

## Expected results

### Example intraoperative case

The outlined methodology has been utilized in one awake-craniotomy as the first participant in an IRB-approved study. For this case, the CyberGlove serial communication was performed using MATLAB. Subsequent measurements will use Python code running on a Raspberry Pi computer. The computer performing the serial communication does not impact the methods of analysis and visualization but using Python running on the Raspberry Pi ensures that the computer performing the communication has no parallel tasks introducing a time lag. In contrast, this could occur with a desktop computer serial communicating with MATLAB.

In this intraoperative case, the surgeon and the participant were told to stimulate or perform the indicated task at an audio buzzer cue, respectively. The hypothesis being tested during this experiment was that different brain stimulation parameters would have different effects on hand task performance. The tasks included single digit flexion, whole hand open-and-closing, F-Socket pinching, or F-Socket squeezing. Depending on response time of the participant, the stimulation might start before or after the task. Before each task, the TTL button was pressed to initiate recording across all the devices. After the task was performed, the button was pressed again. The latencies of each device’s response to the TTL signal are dependent on device sample rate and initiation method. Every device, besides the CyberGlove, has a parallel signal stream containing the TTL pulse which means latencies are calculated as plus or minus one sample (Table 1). The CyberGlove’s response latency was calculated as part of a manuscript in preparation. During kinetic trials (pinch or squeeze), a new TekScan software recording window needed to be opened for every recording or the pulse would not trigger a recording.

**Table 1:** Synchronization method with their mean, range, and 95% confidence interval for each data stream.

	CGIII	F-Socket	GoPros	Stim/Task Cue	Nihon Kohden
Sync method	Start-TTL	BNC-TTL	LED-TTL	Piezosensor-TTL	TTL-ECoG
Task-TTL latency (mean)	94.5 ms	0.0 ms	0.0 ms	0.0 ms	0.0 ms
Task-TTL latency range	66.7 ms	10.0 ms	68.0 ms	0.2 ms	0.2 ms
Task-TTL latency 95% CI	0.4 ms	5.0 ms	34.0 ms	0.1 ms	0.1 ms

Comparison of synchronization method and relative latency (mean +/- 95% CI) of device output to button switch voltage (Task-TTL). The latencies are rounded.

**Fig 2:**
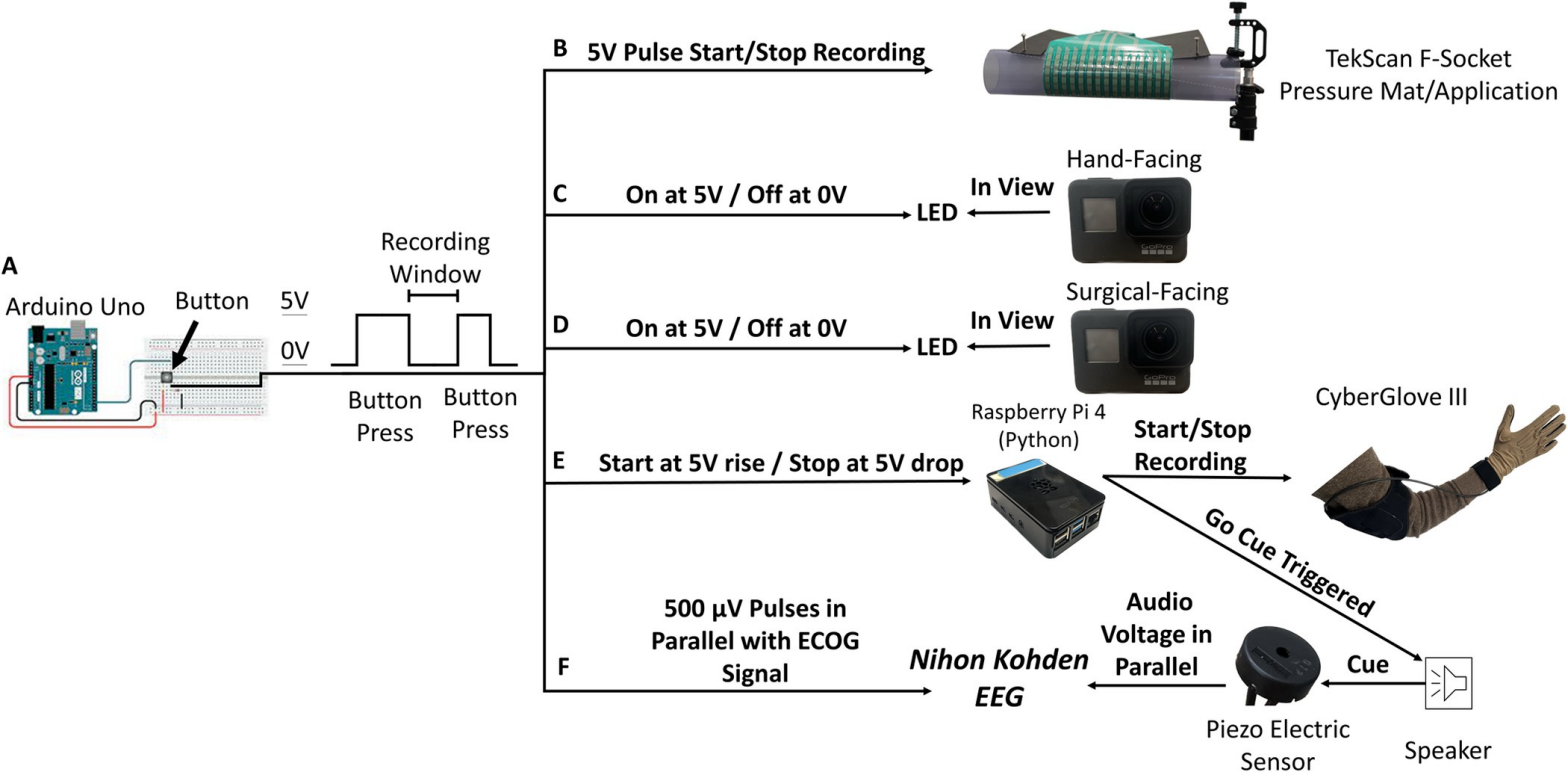
Synchronization diagram. (A) Arduino Uno Rev3 powers (5V) a circuit, which is gated by a button-switch, that varies the voltages across parallel outputs for each device. A task window occurs in between two button presses. (B) TekScan Software reads voltage pulse through BNC Cable plugged into the F-Socket’s VersaTek Hub to trigger the start and end of a recording. (C-D) Voltage pulse/Button press corresponds to LED being ON. (E) Parallel voltage signal is routed back into an Arduino input terminal that is read by MATLAB and used to trigger the CyberGlove start/stop serial commands and an audio go/stimulate cue about three seconds after the button is released. (F) Button-gated voltage is sampled in parallel with ECoG via a Nihon Kohden DC channel. (G) Piezoelectric sensor’s voltage is sampled in parallel via a Nihon Kohden DC channel.

Since every signal (besides the CyberGlove) had a corresponding TTL signal, each task was aligned with the corresponding time window between the TTL-voltage pulses triggered from the start and stop button press (Fig 2). The same time window was extracted from the GoPro video by averaging red intensity in the video around the red LED. The CyberGlove and TekScan data were saved in individual files that corresponded to only the movement windows.

**Fig 3:**
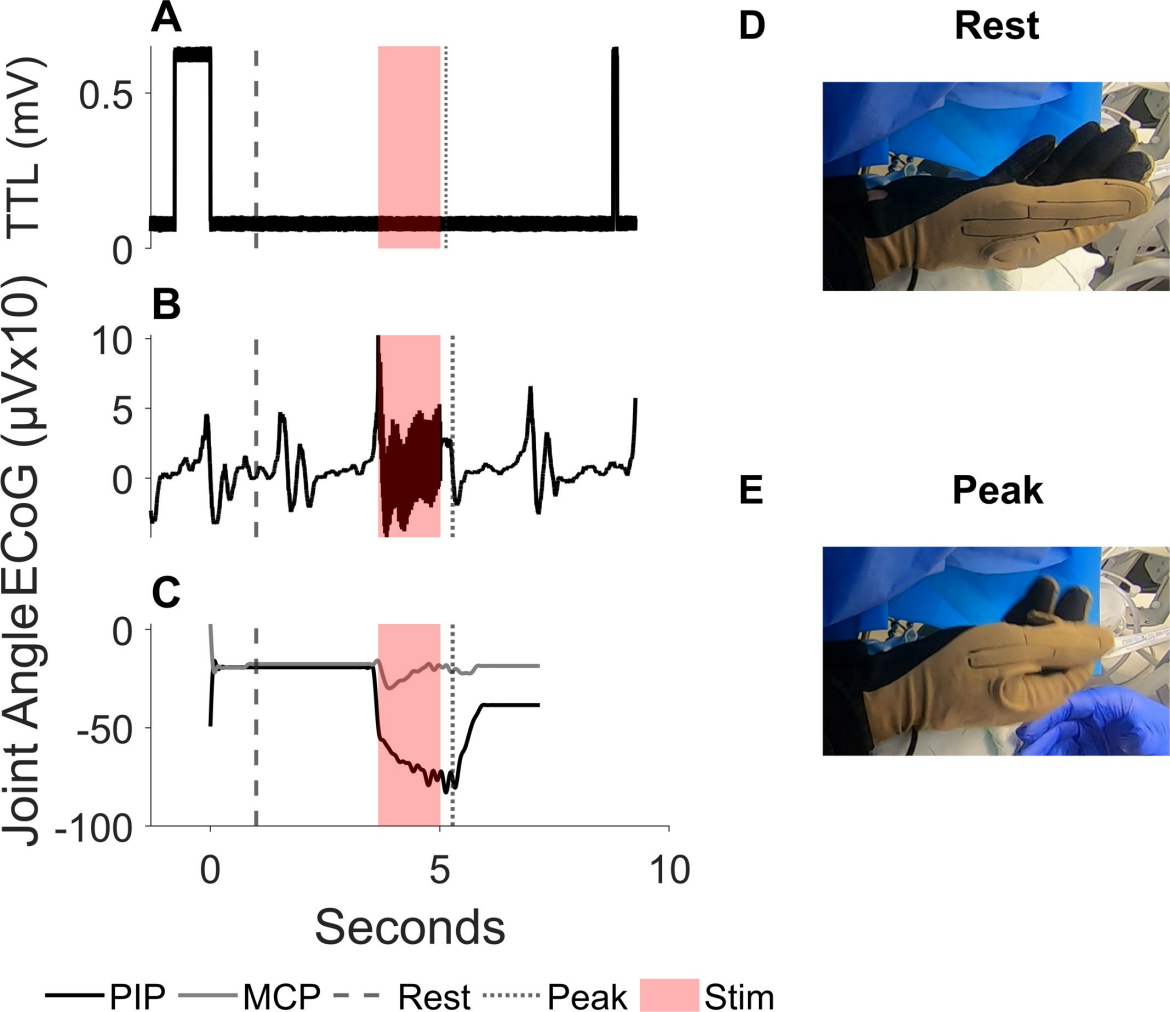
Sample intraoperative kinematic index flexion trial. (A) TTL voltage signal from the button press that initiates and stops recordings. (B) ECoG from one electrode during an index flexion trial in the operating room with a rest frame and peak flexion frame marked. (C) Index metacarpophalangeal (MCP) joint (gray) and proximal interphalangeal (PIP) joint (black) angle data from the index flexion trial with the rest frame and peak flexion frame marked. (D) The GoPro frame of the patient’s hand during the rest frame. (E) The GoPro frame of the patient’s hand during the peak flexion frame.

One index flexion trial with cortical stimulation was chosen as an example of synchronized ECoG and joint angle (Fig 3B and 3C). Simultaneous data from the CyberGlove and the patient facing GoPro camera are shown in Fig 3C–3F for a rest point and the peak point in the movement.

**Fig 4:**
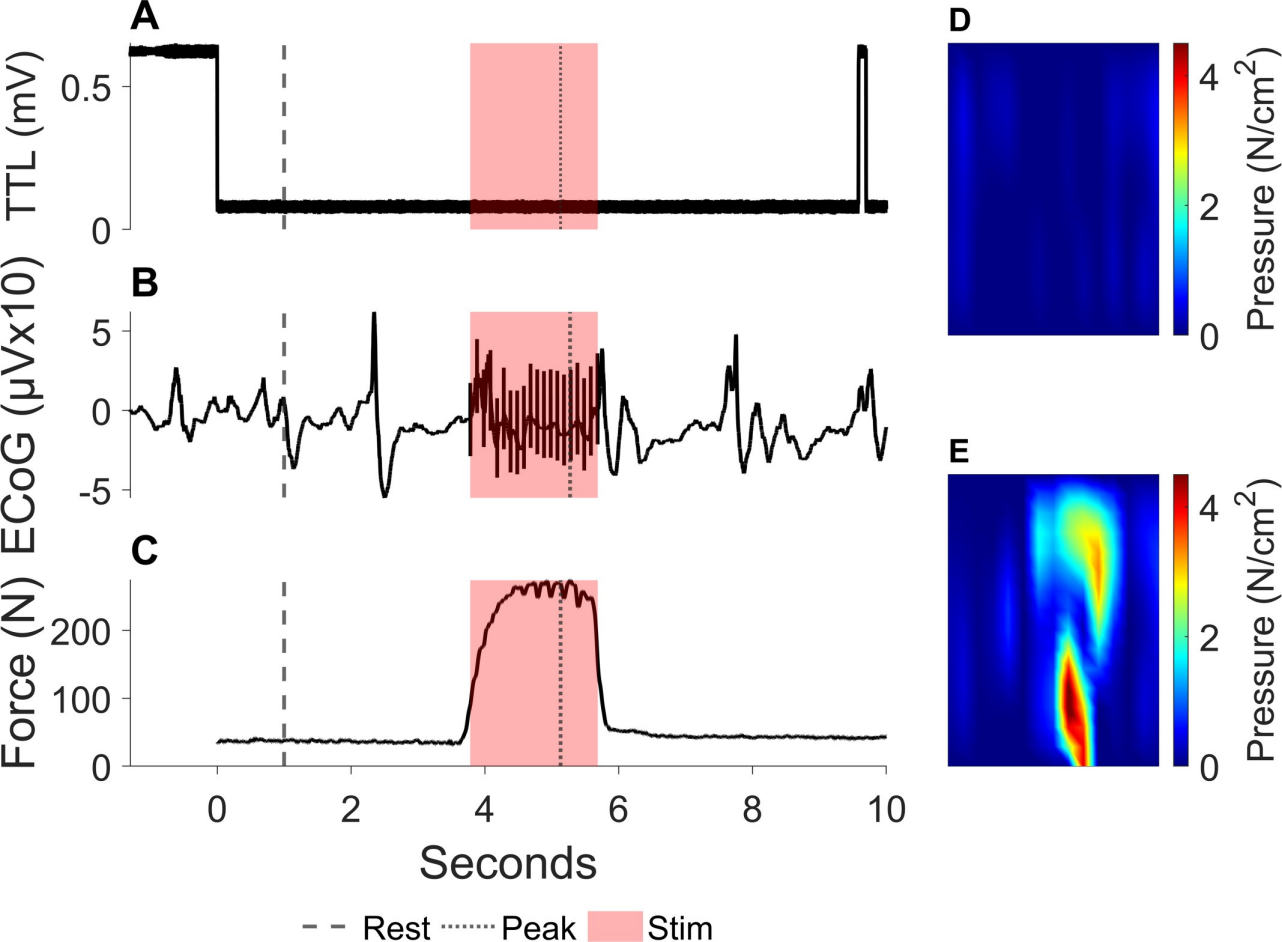
Sample kinetic whole hand trial. (A) TTL voltage signal from the button press that initiates and stops recordings. (B) ECoG from one electrode during a whole hand squeeze trial in the operating room with a rest frame and peak force frame marked. Stimulation period is marked with red box. (C) Force output from the F-Socket during a whole hand squeeze trial in the operating room with a rest frame and peak force frame marked. Stimulation period is marked with red box. (D) F-socket pressure map (15 x 16 sensors/544.8 x 190.5mm) during the rest frame. The top and bottom of the frame shows pressure from the fingers and thumb., respectively. (E) F-socket pressure map during the peak force output frame.

One trial of a whole-hand-squeeze during cortical stimulation is shown in Fig 4 to show force/pressure measurements aligned with ECoG (Fig 4). At rest, the pressure map is blank and the ECoG shows that no stimulation was occurring (Fig 4B and 4C). At the peak of the whole had squeeze, the outline of the pressure applied by the fingers and thumb are visible, and the ECoG shows that applied pressure occurred during cortical stimulation (Fig 4B–4E).

This method allowed for minimal impact on the many other tasks being performed in the operating room. The set-up in the operating room can occur prior to the surgery in around 15 min. Using cable management, normal operating room activity can be unhindered with the operator of the synchronization circuit distant from the patient and the surgeon. In the case shown, the operator was 3 meters away, but the wires can be constructed if necessary to put the operator even farther away.

The optimal method of synchronization for any device is a parallel input stream for the TTL voltage signal for better offline data synchronization. However, the CyberGlove, F-Socket, and cameras do not have that option, thus making the described technique an effective method for synchronizing these signals.

## Supporting information

S1 File Step-by-step protocol, also available on protocols.io. (PDF)

## Abbreviations

OR: Operating Room
DCS: Direct Cortical Stimulation
ECOG: electrocorticography
DoF: Degrees of Freedom
TTL: transistor-transistor logic
CI: Confidence Interval
MCP: metacarpophalangeal
PIP: proximal interphalangeal
