# Supplementary material for: Synchronization of kinetic and kinematic hand tasks with electrocorticography and cortical stimulation during awake craniotomies
Source: PLoS One. 2023 Mar 27;18(3):e0283460. doi: 10.1371/journal.pone.0283460 (PMC10042330; doi:10.1371/journal.pone.0283460)
Supplement: S1 File — (PDF) [file pone.0283460.s001.pdf]

knowledge changing life

CPHNVJ5E

Synchronization of kinetic and kinematic hand tasks during awake craniotomy V.(cphnvj5e)

ltaquet[1], bjconway[1], Timothy F Boerger[1], Sarah C Young[1,2], stschwartz[1], Brian Schmit[2,1], maxkrucoff[1,2]

[1]Medical College of Wisconsin; [2]Marquette University

ltaquet

ltaquet

Protocol Info: ltaquet, bjconway, Timothy F Boerger, Sarah C Young, stschwartz, Brian Schmit, maxkrucoff . Synchronization of kinetic and kinematic hand tasks during awake craniotomy. **protocols.io** https://protocols.io/view/synchronization-of-kinetic-and-kinematic-hand-task-cphnvj5eVersion created by ltaquet

Created: Feb 15, 2023

Last Modified: Feb 15, 2023

PROTOCOL integer ID: 77070

Keywords: direct cortical stimulation, hand kinematics, hand kinetics, hand function

ABSTRACT

Title: Synchronization of kinetic and kinematic hand tasks with electrocorticography and cortical stimulation during awake craniotomies

Introduction

Intraoperative direct cortical stimulation (DCS) is standard of care to verify functional-anatomical relationships of critical brain areas during awake craniotomies. Quantizing general measures of kinetic and kinematic motor responses would minimize subjective fluctuation and improve mapping confidence. Furthermore, there is enormous opportunity for scientific inquiry into motor neurophysiology during these cases if the data is carefully collected and synchronized.

Objective

Methods for quantizing motor responses during awake craniotomies must aim to minimize disruption to operating room (OR) staff and the procedure. The methodology defined here aims to allow for the synchronized assessment of whole hand kinematics, kinetics, DCS, electrocorticography (ECOG), surgical video, and task video.

Methods

We use a high-resolution force mat, a 23-degree-of-freedom (DoF) data glove, a standard clinical Nihon Kohden, and a custom synchronization platform to collect our data. Synchronization of the devices in the intraoperative set-up relies on a shared button-gated TTL voltage signal where the data stream for a task is marked or initiated a button press. MATLAB and Python are used for post-processing of the data streams.

Results

Each recording on-set was assessed for lag from a button-press initiation to data being recorded. The 95% lag confidence interval for whole hand kinematics, kinetics, DCS, electrocorticography (ECOG), surgical video, and task video streams were 95.3-95.7 ms, 0.0-5.0 ms, 0.0-0.1ms, 0.0-34.0, and 0.0-34.0 ms, respectively.

Conclusion

This methodology allows for minimal impact on the many other tasks being performed in the operating room. The set-up in the operating room can occur prior to the surgery and quickly. Using cable management techniques, normal operating room activity can be unhindered with the operator of the synchronization circuit at a distance. The data produced was reliably synchronized and can provide important insights into motor neurophysiology.

MATERIALS

Devices

- Nihon Kohden EEG-1200AA series (Any Clinical EEG with parallel DC channels)
- CyberGlove III (CyberGlove Systems)
- TekScan F-Socket, VersaTek Hub, and TekScan Software
- Arduino UNO Rev3 Starter Kit (contains Arduino, resistors, breadboard, push button gates, pezioelectric sensor, USB-Mini USB)
- Canakit Raspberry Pi 4 Starter
- Stock GoPro Hero7
- PeauProductions Custom GoPro Hero 7 Black with 8.25mm (47mm) f/2.8 lens

Circuit Manufacturing

- Prototyping Board
- Screw Terminal Block to BNC (TekScan)
- Wire Stripper
- 2x Screw Terminal Block to Male 1/8" Jack
- 2x 2.5mm Male to 3.5mm Female Stereo Audio Adapter
- 22 Gauge 2 Conductor Electrical Wire, 20M/65.6ft Flexible Black PVC Jacketed Hookup Wire, 22 AWG Tinned Copper

Operating Room Set-up

- 20ft USB-C Cable (Go Pro)
- GoPro Mounting Clamp
- Velcro cinch straps (cable management)
- Sametop Frame Mount Housing (GoPro)
- 2.5 inch PVC Pipe
- 2x CAMVATE Universal C-Clamp for Desktop Mount Holder with 1/4"-20 & 3/8"-16 Thread Hole - 1121
- EMART Light Stand, 6.2ft Photography Stands (2 Pack)
- Speaker (externally powered; Battery-powered speakers cut-off the beginning of sounds due to sleep functions)
- Aux Cable
- Media Cart

Project Steps

The major steps to replicating the methodology for synchronization of hand kinetics and kinematics during awake cortical mapping:

- Obtaining circuit components and recording devices
- Assembling circuit
- Downloading MATLAB code and Initializing Local Environment for Data Processing
- Downloading Python code and Initializing Raspberry Pi 4 Environment for Recording
- Post-processing

Required Skills

Electronics assembly, soldering, MATLAB programming

Constructing Synchronization Circuit

4w

1 **Obtaining Circuit Parts and Devices**

The TekScan and CyberGlove system devices can be procured directly from the companies. Any clinical EEG device used for seizure monitoring during an awake craniotomy and cortical mapping can be used if it has at least two parallel DC channels. Using a different EEG device may change the input jack size needed for connecting to the TTL voltage. The custom GoPro was sourced from PeauProductions.

All parts besides the previously mentioned devices can be sourced from Amazon, Digikey, Canakit, or B&H Photo.

2 Using a breadboard or perfboard construct the circuit diagramed below:

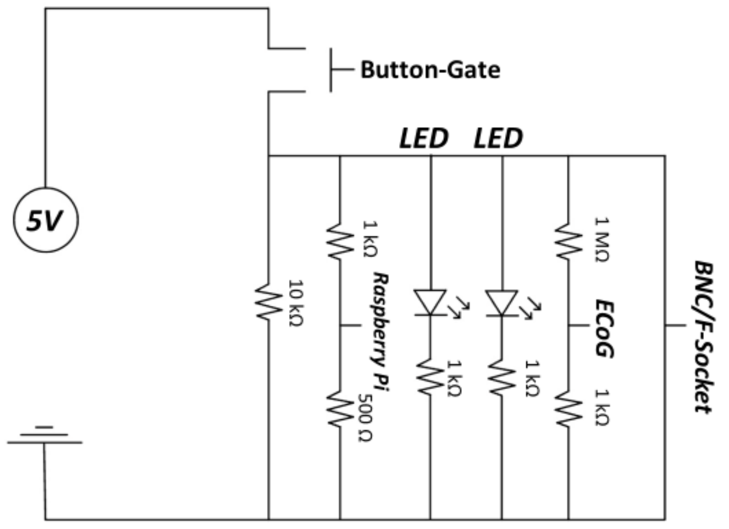

Button-gated voltage TTL circuit diagram:
- Ensure long enough wire segments are used to connect to devices without obstructing OR traffic. We made the wires for each device ~25 feet long to allow for running the wire along OR booms and light arms.

3 To allow for each device to be connected and disconnected after a recording, construct circuit-device connecting wires with Molex connectors for circuit connections, device specific input connector, and 22-gauge two conductor electrical wire.

Device Specific Input Connector:

TekScan VersaTek Hub - BNC
Nihon Kohden DC Channel - 1/8in Audio Jack
Patient-Facing LED - LED soldered to wire
Surgical-Facing LED - 2-terminal screw-terminal soldered to wire

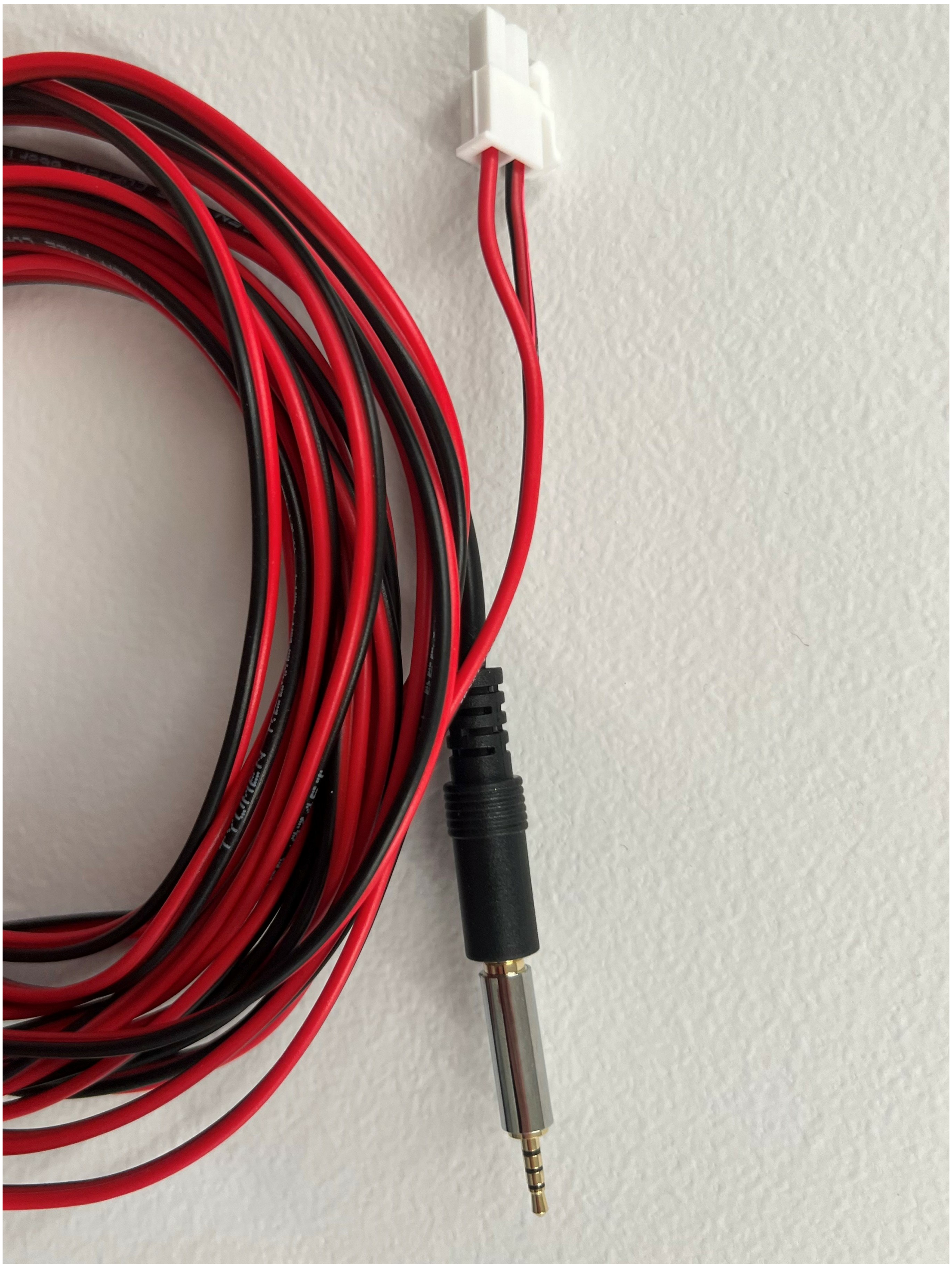

Two conductor electrical wire with Nihon Koden DC jack and Male Molex Connector

4 Construct the piezoelectric sound sensor by soldering the sensor terminals to one end of a 22-gauge two conductor electrical wire and attach an 1/8in audio jack to the opposite end. The piezoelectric sensor produces its own voltage with sound waves to detect cues and does not need a power source.

5 Mount an LED to the custom GoPro in a way that does not interfere with the mounting case and

shows the LED in the corner of its field of view when recording.

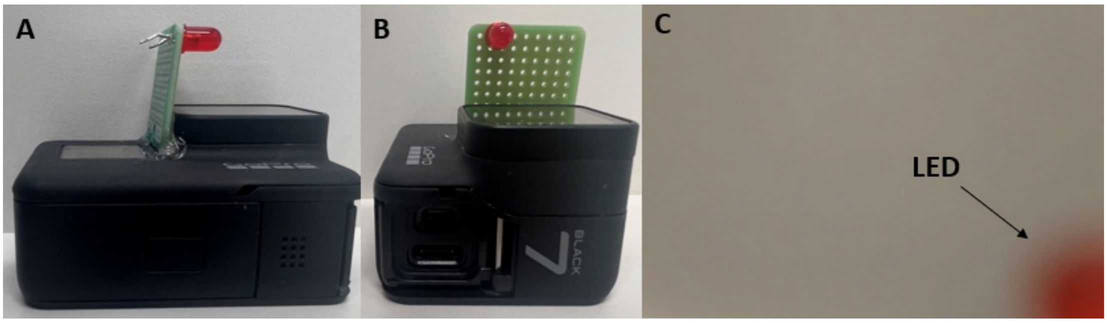

A: GoPro-LED Set-up Bottom View
B: GoPro-LED Set-up Side View
C: GoPro Field-of-View

Establishing Python and MATLAB Environment

1h

6 Download linked GitHub repository for CyberGlove data acquisition Python code run on the Raspberry Pi 4 and the CyberGlove data processing and calibration MATLAB code. Migrate the Python code and directory configuration to the Raspberry Pi 4. Migrate the MATLAB code to the location for data processing.

MATLAB CyberGlove Data Processing:
https://github.com/ltaquet/CyberGloveDataProcessing

RPi CyberGlove Data Acquisition: https://github.com/ltaquet/RPi_CGIII

Data Acquisition Set-up

10m

7 Attach female Molex connectors to corresponding male Molex connectors for all devices, and connect circuit to 5V power supply and ground. Attach the piezoelectric sensor to the table in front of the speaker. Connect both the TTL and piezoelectric audio jacks to the clinical EEG's DC channels, ensure that those channels are on and recording.

8 Mount custom GoPro on the surgical lights with clamp mount. Mount normal GoPro on tripod with patient hand and LED in frame. Use Velcro straps to run charging cable and LED-Circuit write along light and boom arms to minimize interference with OR activity.

9 Turn on CyberGlove and plug-in USB cable connecting the Pi and the glove. It is best to put the CyberGlove on the patient when they arrive in the operating room before surgery and while they are awake, but wait to turn on the glove until the recordings are about to be done.

10 Connect to the Raspberry Pi via SSH. After ensuring a data directory named with the participants PID exists, navigate to 'Documents/code/cg3' and run cg.py with the line 'python3 cg.py *PID*'. Wait to trigger a task until it prompts READY.

```
ltaquet@raspberrypi:~ $ mkdir Documents/data/MCW001
ltaquet@raspberrypi:~ $ cd Documents/code/cg3/
ltaquet@raspberrypi:~/Documents/code/cg3 $ python3 cg.py MCW001
Got fix: 2022-07-12 10:53:16.768322
10:53:43.003829
b'!!G\x03?pgZESOLC\x99^>]yW;D\x92\x01\xe6h\x001ds\x001eu\x001dw\x001u\x01\x001m\x03\x001ts(HH:MM:SS)\r\n10:53:42\x00'
10:53:43:13
READY
```

Terminal view for initialization and start of a CyberGlove recording using Python on a Raspberry Pi

11 If performing a kinetic task using the TekScan, open the F-Socket Research Application. Ensure the F-Socket is properly connected and registered by the application. Enable BNC trigger for start and stop of recording, and set recording time to max allowed. Zero the pressure mat without the participant's hand on it. Save the zeroing calibration file.

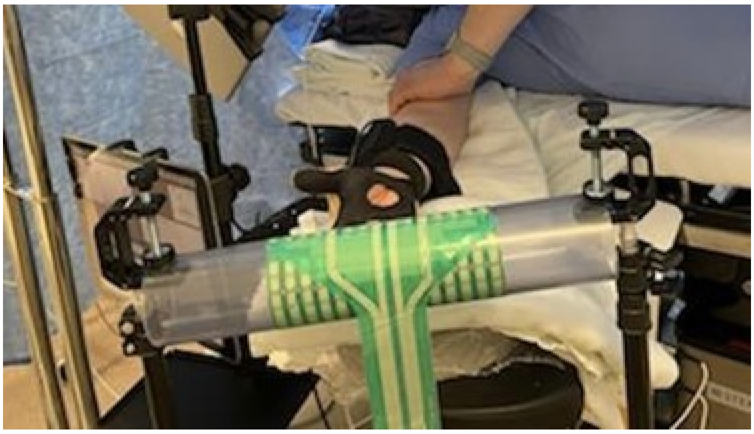

Placement of TekScan F-Socket Kinetic Task Set-up

Data Acquisition

12 **Recording a Task**

Participant should have a visual cue for the task to be performed in their visual field and have been prepped pre-op.

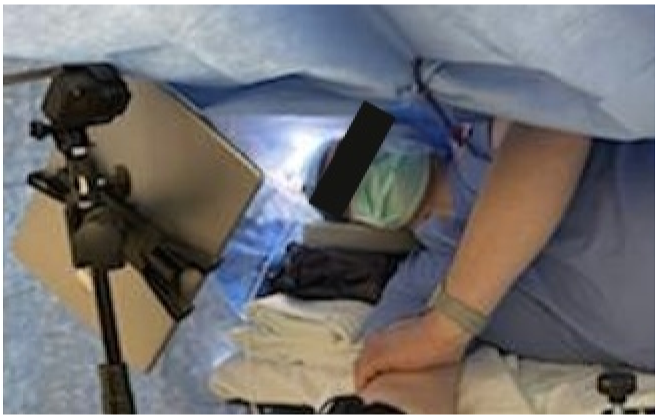

Example Participant with a display in view for visual cues

12.1 **Kinematic Task**

Press and release TTL Button. Tone from the speaker cues the participant and the surgeon for when the task and stimulation (if performed) should occur. Press TTL Button again to stop.

12.2 **Kinetic Task**

Open a new recording file in the F-Socket Research application. While holding down the TTL Button, press record in the app. Release the TTL Button when ready to trigger task. Tone from the speaker cues the participant and the surgeon for when the task and stimulation (if performed) should occur. Press TTL Button again to stop

Data Processing: CyberGlove

1h

13 Before analyzing a new participant, run newPatient.m (Code/CyberGloveDataProcessing) and answer prompts being sure to use the same PID (case-sensitive) as the PID directory on the Raspberry Pi. It is easiest to have a PID and PID**L** to differentiate left and right handed recordings.

14 Copy all of the raw CyberGlove data from "Documents/data/*PID*" in the Raspberry Pi to "Patients/*PID*/Raw Data".

15 Run readBinCGLoop_rasp.m and answer prompts. This will create an individual task directory in 'Raw Data' and move the processed binary data into it. The raw data will be converted into a ".MAT" file containing uncalibrated CyberGlove data for all of the sensors.

16 Run ProcessRawCGmat.m and answer prompts. This will create an individual task directory in 'Uncalibrated Data'. The Uncalibrated data will be converted into a ".MAT" file containing calibrated CyberGlove data (both filtered and unfiltered degrees and radians) for all of the sensors, raw data, CyberGlove timestamps for each sample, and number of dropped samples. Each file should correspond to the period in between TTL Button presses.

Data Processing: TekScan

30m

17 After recording, apply the saved zero calibration file to all of the recordings. Saving each file as an '.CSV' allows for MATLAB processing. Edit Intraop_Process_ASCII.m to match your directory layout and desired task file names. Run Intraop_Process_ASCII.m to convert the CSV ASCII file into a 4D Matrix '.MAT' file.

Data Processing: EEG + DC Signals

30m

18 If a Nihon Kohden was used to record the ECoG, piezoelectric sensor, and TTL signals, unpack and process the session folder in the MATLAB package 'BrainStorm' (Tadel F et al, 2011). Use BrainStorm to export the parallel signal arrays into '.MAT' files.

Data Processing: GoPro

1h

19 Edit the video for each task into sections that contain video before and after the LED flash from the TTL pulse. Run FindLEDDrop.m with the video file of interest loaded into a MATLAB video object to find the frame number that corresponds to the falling edge of the TTL pulse.

Data Processing:Aligning Streams

2h

20 All data for a task can now be aligned with the TTL pulse drop at the start of a task. The CyberGlove and TekScan files starts correspond to falling edge of the TTL pulse. If the order that tasks were recorded, a corresponding file from these modalities can be aligned with ECoG and the piezoelectric sensor signal with the parallel TTL signal. The frame number of the LED drop can be used to align the GoPro video to these signals.
